# SPRM: spatial process and relationship modeling for multiplexed images

**DOI:** 10.1093/bioadv/vbag019

**Published:** 2026-01-21

**Authors:** Ted Zhang, Haoran Chen, Young Je Lee, Matthew Ruffalo, Robert F Murphy

**Affiliations:** Ray and Stephanie Lane Computational Biology Department, School of Computer Science, Carnegie Mellon University, Pittsburgh, PA 15213, United States; Ray and Stephanie Lane Computational Biology Department, School of Computer Science, Carnegie Mellon University, Pittsburgh, PA 15213, United States; Ray and Stephanie Lane Computational Biology Department, School of Computer Science, Carnegie Mellon University, Pittsburgh, PA 15213, United States; Ray and Stephanie Lane Computational Biology Department, School of Computer Science, Carnegie Mellon University, Pittsburgh, PA 15213, United States; Ray and Stephanie Lane Computational Biology Department, School of Computer Science, Carnegie Mellon University, Pittsburgh, PA 15213, United States

## Abstract

**Motivation:**

There has been tremendous recent growth both in technologies for measurement of many different markers in the same tissue and in resulting datasets (especially from projects such as HuBMAP and the Human Cell Atlas). Analysis of images in these datasets is often restricted to measuring the amount of each marker in each cell. While this is important, it ignores other information that is contained in tissue images. SPRM was therefore created for use in the HuBMAP image analysis pipelines and can be used for any spatial proteomics dataset.

**Results:**

It calculates a number of measures of image quality, including metrics for the quality of cell segmentation, and extracts many different types of cell features that give much richer characterization than just marker intensities per cell. Different feature types are used to cluster cells into potential cell types to view the tissue through these different lenses, and these are compared to expert annotations if provided in order to define cell subtypes. The package also constructs a cell adjacency matrix to characterize cell spatial distributions. Example analyses are provided in Supplementary Information.

**Availability and implementation:**

SPRM is available as python open source at https://github.com/hubmapconsortium/sprm and as a PyPI package.

## 1 Introduction

In the past few years, the development of new technologies has made potentially achievable a previously unattainable goal: to identify all cell types in the human body. Projects such as the Human Cell Atlas (Regev *et al*. 2017) and the Human BioMolecular Atlas Program (HuBMAP) (Snyder *et al*. 2019, Jain *et al*. 2023) have large scale data collection efforts using single cell sequencing and a range of tissue imaging methods. A hallmark of the new tissue imaging technologies is the ability to image large numbers of macromolecules in the same sample, often through multiplexed imaging (Moffitt *et al*. 2022). These produce multichannel images in which each channel measures the spatial distribution of a distinct molecule, such as a transcript, protein or metabolite. The availability of large numbers of images through these and other projects creates a need for tools that can efficiently extract useful quantitative features from large, complex tissue images. Previously described tools, such as Cytokit (Czech *et al*. 2019) largely focused on image preprocessing, cell segmentation, and cell intensity quantitation. Less attention has been paid to more detailed analysis of the spatial information contained in multiplexed images. To address this need, we have developed SPRM for use in the image processing and analysis pipelines of the HuBMAP project, as well as for routine use for any spatial proteomics dataset [it is also currently used in the Cellular Senescence Network (SenNet) project (SenNetConsortium 2022)]. In addition to the full program, we anticipate that some of the specific analyses performed by SPRM may be useful for incorporation into other analysis pipelines. We have therefore created a python package that provides modules for individual analyses.

## 2 Methods

SPRM is written in python 3 and does not require any GPU resources. It is optimized to make use of available parallelization.

**Figure 1:**
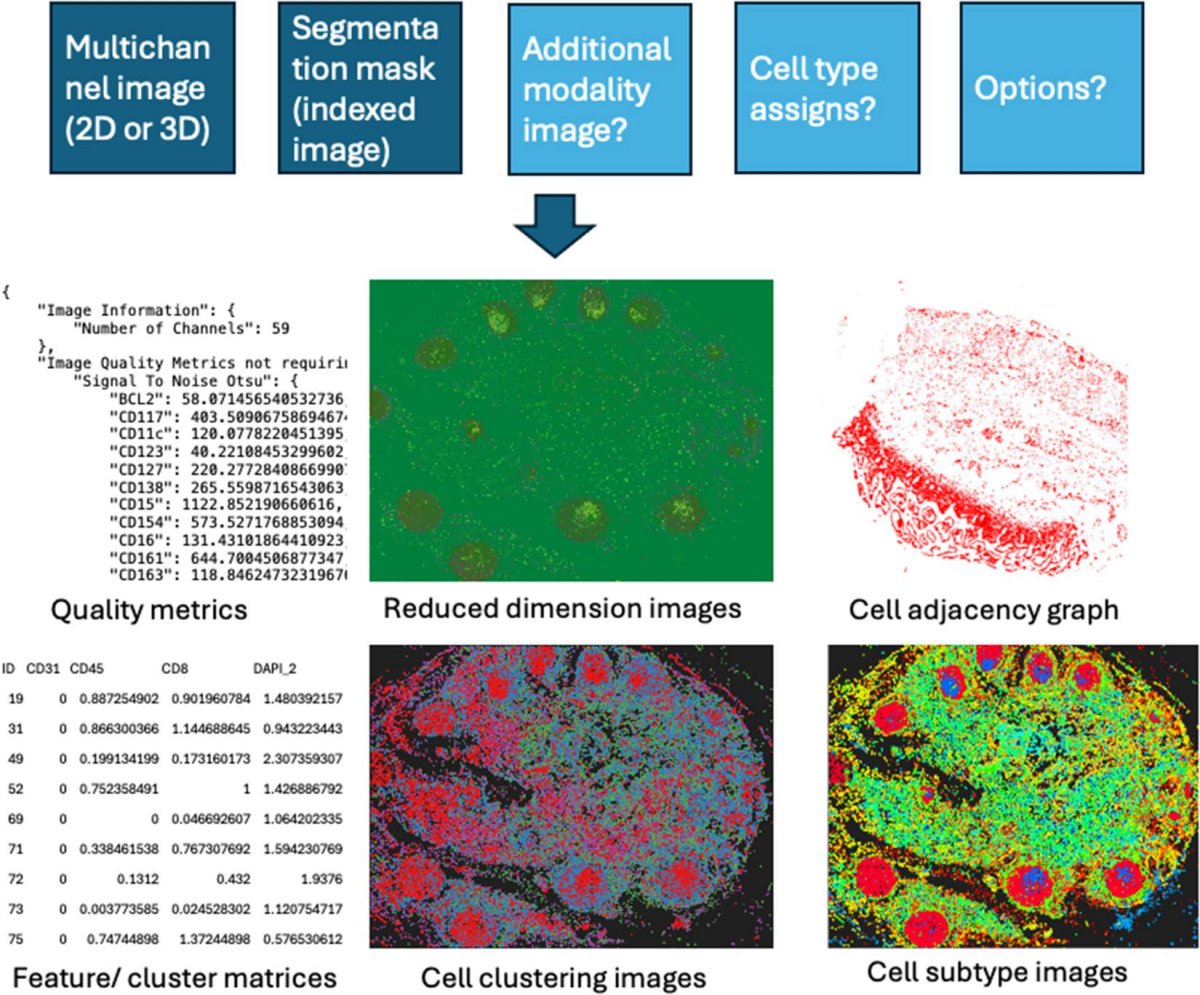
SPRM flow diagram.

Figure 1 provides an overview of the capabilities provided by SPRM. These are summarized below, and detailed descriptions and examples can be found in the online Supplementary Information. These capabilities are modular and can be turned on or off through the options file. Some modules have parameters that affect their behavior, and the default values for these can also be changed through the options file.

The two required inputs are a multichannel OME TIFF image file containing intensities for multiple markers, and a multichannel OME TIFF indexed image file containing four segmentation masks for each cell. These mark pixels in the whole cell, the nucleus, the cell boundary, and the nuclear boundary. The segmentations can be produced by any desired method, but must be converted, if necessary, into this format. The two inputs can be specified as separate paths corresponding to multiple files to be processed, in which case filenames must correspond between the folders. Input images can be multicolor 2D (C by Y by X) or multicolor 3D (C by Z by Y by X).

Outputs are described below and can be obtained either by running the full SPRM program or by using specific modules from the SPRM package.

## 3 Results

### 3.1 Quality measures

The first major focus of SPRM is to enable evaluation of the quality of acquired images and of provided cell segmentations. The main purpose of these metrics is to permit image quality information to be taken into account in subsequent analyses. For example, they might be used to exclude images below a certain quality threshold from further analysis, or to weight results from a collection of images by the quality score of each image. They can also potentially be used to monitor for problems with sample preparation, reagent batches, or instrument performance. The quality metrics are described in detail in Fig. 2, available as supplementary data at *Bioinformatics Advances* online. The main metrics measure image quality as the strength of signal to background. These are reported for each channel by three methods. The first two estimate *pixel* level background either by the Otsu method or by the standard deviation of the channel intensity. The third measures the ratio (for each channel) of the average *cell* intensity to the average background intensity. The average ratio of nuclear to cell intensity is also reported, which may reflect how well the images reflect expectations for a given marker (i.e. whether it is primarily in the nucleus or not).

In addition, potentially useful measures such as the fraction of image occupied by cells, the total number of cells, and the total intensity in each channel are reported. Lastly, scores for the quality of cell clustering into different numbers of cell types are reported.

Independently, a set of metrics that reflect the quality of the provided cell and nuclear segmentation are produced. They are based upon assumed properties of good segmentations and are used to calculate an overall quality score that does not require any human input. They have been described previously for both 2D (Chen and Murphy 2023) and 3D (Chen *et al*. 2025).

Example analyses shown in Figs 8 and 9, available as supplementary data at *Bioinformatics Advances* online using the image quality metrics reveal variation among different HuBMAP images from different tissues.

### 3.2 Reduced dimension summary images

The number of markers typically measured in spatial proteomics is too large to display in a single image. To provide an approximate overview of the spatial relationships of all markers, SPRM produces three images that show different lower-dimensional representations of the pixel spectra. The first uses the first three principal components and the second uses non-negative matrix factorization. The third uses k-means clustering of the pixel values to choose three “pixel types” and assign each to a different color. An example is shown in Fig. 3, available as supplementary data at *Bioinformatics Advances* online.

### 3.3 Cell adjacency graph

In order to allow comparison of the spatial arrangements of cell within different images, SPRM produces cell adjacency matrices (Fig. 3, available as supplementary data at *Bioinformatics Advances* online). Cell adjacency is often based upon cell centroids, which ignores cell size and shape. In contrast, SPRM records which cells are within a specified number of dilations of each cell’s actual boundaries. Analysis of cell adjacency graphs shown in Fig. 11, available as supplementary data at *Bioinformatics Advances* online reveal differences in cell arrangements within and between tissues.

### 3.4 Feature values

The central function of SPRM is to generate features describing different aspects of each cell in an input image. The types of features are described in the Supplementary Information. The first consists of (for each channel for each cell), the mean and total intensity of the whole cell, nucleus, cell boundary, and nuclear boundary. The second consists of the covariance matrix of the channel intensities of each cell, which reflect differences in the spatial distribution of each marker within each cell. The third is cell shape descriptors in the form of coordinates in a lower-dimensional cell shape distribution [see (Ruan and Murphy 2019, Chen and Murphy 2023) for description and comparison of various cell shape analysis methods].

### 3.5 Cell typing and subtyping

Using various combinations of the feature subsets, SPRM makes various unsupervised estimates of the cell types present in a given image. These can be used for visualization directly. As described in Supplementary Information, use of a diverse set of cell features reveals differences in cell clusters/types beyond those revealed by marker intensities alone (Fig. 6, available as supplementary data at *Bioinformatics Advances* online). The clusters can also be compared to results from externally produced cell type annotations. Such cell type annotations are usually produced using only per cell marker intensities from a specific set of markers and thus do not make full use of the markers in a particular image. Therefore, if provided, SPRM finds the unsupervised clustering that yields the best many-to-one match with the provided cell types and then identifies potential subtypes of each cell type (Fig. 7, available as supplementary data at *Bioinformatics Advances* online).

## 4 Discussion

We have developed SPRM to aid in processing, interpretation and evaluation of multiplexed images, especially from spatial proteomics. In particular, SPRM provides metrics that allow estimation of the relative quality of different tissue images. A thorny issue is establishing how well such metrics meet their desired goal, and the typical approach is to compare with results from human experts. However, it is not always clear who such experts would be and how well matched the task is to human abilities. In addition, significant variation is observed between different experts. In the case of the cell segmentation, the metrics calculated by SPRM have been shown to capture the superior performance of highly trained automated segmentation methods compared to human experts (Chen and Murphy 2023). The principle successfully applied in that case is to identify the characteristics expected of a good segmentation and design metrics to capture. We have applied this principle to design image quality metrics (reflecting well accepted measures such as signal-to-noise ratios) and used them to reflect differences between collected images from different tissues. We anticipate that future work may generalize this approach to capture more characteristics, such as compartment or cell edge sharpness.

SPRM provides a different feature set to describe the level and pattern of markers in each cell. We have used these to cluster cells into potential cell types and compared the results to externally generated cell type assignments. Future work will be needed to establish the significance of differences in cell types and subtypes observed using different feature sets.

## Supplementary Material

vbag019_Supplementary_Data

## Data Availability

The data and software underlying this article are available in Github at https://github.com/murphygroup/SPRM-RRA.
