# Supplementary material for: SPRM: spatial process and relationship modeling for multiplexed images
Source: Bioinform Adv. 2026 Jan 21;6(1):vbag019. doi: 10.1093/bioadv/vbag019 (PMC12895062; doi:10.1093/bioadv/vbag019)
Supplement: vbag019_Supplementary_Data [file vbag019_supplementary_data.pdf]

Supplementary Information and Documentation

SPRM: Spatial Process and Relationship Modeling for Multiplexed Images

Ted Zhang, Haoran Chen, Young Je Lee, Matthew Ruffalo, and Robert F. Murphy*
Ray and Stephanie Lane Computational Biology Department, School of Computer Science, Carnegie Mellon University
Pittsburgh, Pennsylvania, United States

*Corresponding author: murphy@cmu.edu

Implementation

SPRM is written in Python (3.8 or newer) and is available from https://github.com/hubmapconsortium/sprm. It uses aicsimageio, an open source package developed by the Allen Institute for Cell Science, to read OME-TIFF images, uses parallelization techniques for multithreaded processes, and OpenCV packages for image processing and analysis. It is compatible with Intel, Apple ARM and AMD Rizen chip sets.

A demonstration shell script and jupyter notebook are provided in the repository as well as an example image and cell mask (see “Simple illustration” in the README).

Individual analyses can also be performed using specific modules in the SPRM package available from PyPI.

A Reproducible Research Archive containing all code and results for the analyses described below is available at https://github.com/murphygroup/SPRM-RRA.

SPRM Inputs

SPRM requires two primary inputs: a path to multichannel image files and a path to corresponding files containing segmentation masks for each cell (Supplementary Figure 1). They should contain masks for the cell and nucleus, but can contain additional cell boundary and nuclear boundary masks. The names of the files in all specified paths (including the optional paths below) for a given sample must match, and images can be two- or three-dimensional (note that throughout the below we use pixel to refer to either pixel or voxel as appropriate). There are four optional inputs that can be included: an output path (default is the current path); an additional path to files containing images of the same samples but from a different modality at lower resolution; an additional path to files containing cell type annotations; and a text file containing options to control various aspects of the processing. The options and their default values are shown in Supplementary Table 1.

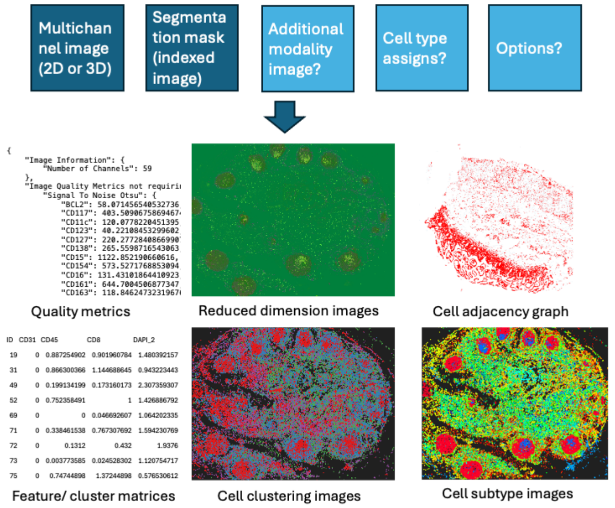

Supplementary Figure 1. SPRM inputs and outputs.

Supplementary Table 1. SPRM options. These options are supplied through a text file.

Options	Default (0/1=F/T)	Description
**General/runtime controls**		
debug	0	Enable verbose/diagnostic mode. Also disables subprocess isolation for multi-image runs.
image_analysis	1	Perform the main image feature-extraction and analysis steps.
**Dimensionality selection and preprocessing**		
image_dimension	2D	Processing mode: 2D or 3D. The default for 3D input images is to select one z-slice to process (see option zslices). Therefore, image_dimension must be set to 3D to process all slices.

zslices	0	For image_dimension=2D, specifies which z-slice to process (setting to 0 picks the slice with the highest total intensity). Ignored for image_dimension=3D.
skip_empty_mask	1	Skip images with no valid cells in the mask.
interior_cells_only	1	Use only interior cells for downstream feature computation (i.e., remove cells touching image edge)
valid_cell_threshold	10	Minimum pixel count for a cell to be considered valid.
normalize_bg	1	Apply background normalization across channels before feature extraction.
**Cell shape analysis**		
run_outlinePCA	1	Find cell outlines and use PCA to derive shape features.
num_outlinepoints	100	Number of points used to sample from cell outline polygons prior to PCA.
**Clustering (cells/voxels/markers)**		
zscore_norm	1	Apply z-score normalization to features prior to clustering/embedding.
tSNE_all_preprocess	none	Define desired preprocessing for tSNE: “none” (don’t zscore), “zscore” (zscore all features together), or “blockwise_zscore” (zscore each block of features (cov/total/meanAll/shape) separately.
num_cellclusters	silhouette 3 10 1	Control choice of number of cell clusters found by k-means: must begin with method (only method currently supported is silhouette) followed by min and max k followed by keep (1=output clustering resuts for each k).
num_shapeclusters	silhouette 3 6 1	Control shape clustering (same structure as num_cellclusters but different default max).
num_voxelclusters	3	Number of clusters for voxel-level grouping (e.g., intensity/region partitioning).
num_markers	3	Number of markers to include for marker-driven analyses/plots.
**Dimensionality reduction (tSNE and PCA before tSNE)**		
tSNE_num_components	2	Number of dimensions for tSNE output.
tSNE_all_tSNEInitialization	pca	Perform PCA transform before tSNE.
tsne_all_svdsolver4pca	full	PCA SVD solver when using PCA init: full or randomized (auto-retries if full fails).

tSNE_all_perplexity	35	tSNE perplexity hyperparameter.
tSNE_all_ee	default	tSNE early exaggeration. default sets it to roughly N/10; numeric values are accepted.
num_channelPCA_components	3	When creating pixel/voxel-wise colored images, number of PCA or k-means components to choose.
**Cell adjacency graph and neighborhood**		
cell_graph	1	Build the cell adjacency graph and related sparse distance matrices.
cell_adj_parallel	0	Use parallel/numba-optimized windowing for adjacency (1) or standard Python (0).
cell_adj_dilation_itr	3	Number of binary-dilation iterations done before checking neighbors.
adj_matrix_delta	3	Padding (in pixels) to add around each cell's bounding box. For efficiency, only pairs of cells with overlapping padded bounding boxes are checked for potential neighbors.
**Reallocation/refinement controls**		
reallocation_descent_rate	0.1	Step size for iterative membership/label reallocation during post-cluster refinement.
reallocation_quit_criterion	0.0001	Convergence tolerance for reallocation iterations.
num_of_reallocations	1	Maximum number of reallocation passes.
reallocation_avg_bg	1	Include/weight background averaging during reallocation updates.
**Evaluation, visualization, and miscellaneous**		
sprm_segeval_both	2	Segmentation evaluation mode; compute metrics for multiple mask channels/variants.
apng_delay	100	Delay (ms) between frames for generated animated PNGs.
subtype_thresh	0.1	Threshold for assigning cell subtypes from provided labels/scores.

The cell segmentation masks may be produced by any of a number of methods (see Chen & Murphy (1) for evaluation of a number of methods), but there must be a corresponding nuclear mask for each cell mask whose indices must be the same. In case a particular segmentation method does not always produce matched masks, a

precursor “mask repair” program can be run before running SPRM (1). This precursor step modifies the mask files by removing any objects in the cell or nuclear masks that do not match and selecting the largest nuclear object if more than one is present for a given cell. It will also trim the nuclear masks to remove any pixels on or outside of the cell membrane of their corresponding cell mask. If masks for the cell boundary and the nuclear boundary are not present, they will be generated. The masks used in the analyses below were created using Cytokit v0.1.1.

Depending on whether the input images are three-dimensional, options may be provided to specify a subset of z slices for a given image dataset. This is especially relevant for (CODEX) datasets since while these are acquired as a stack of 2D images but many of these are out of focus.

SPRM Methods and Example Outputs

SPRM places files of various types containing the outputs described below into the specified output folder. File names are generated by appending a brief description of the contents onto the base name of the input file. These files can be grouped and displayed using the provided jupyter notebook “visualizeSPRMoutputs.ipynb”.

Segmentation Quality Metrics

A critical measure of image quality is how well the image can be segmented into single cells. SPRM therefore reports segmentation quality metrics and an overall segmentation quality score using the CellSegmentationEvaluator package (https://github.com/murphygroup/CellSegmentationEvaluator) (1). The metrics are based on a series of assumptions about desired characteristics of a good segmentation, such as consistency of marker expression with a cell type.

Preprocessing

After the segmentation quality metrics are calculated, SPRM performs preprocessing to prepare for subsequent analysis. Any cells in the mask that are touching an edge of the image are removed, since accurate quantification cannot be achieved for these partial cells. Then, for each channel a background intensity is found (as the average of all pixels outside cells) and channel intensities are normalized to signal to noise ratios by dividing each channel by its respective background level. This facilitates comparison of channel intensity values between images that may have been acquired with different settings.

Image Quality Metrics

In addition to the segmentation quality score, a very important output from SPRM is a JSON file containing quality metrics for each image (see Supplementary Figure 2). As we did for the cell segmentation metrics, we designed the quality metrics based on assumptions about the characteristics of high quality tissue images. The first type does not require background segmentation. These consist of the fraction of total image area/volume occupied by cells, and three sets of metrics of channel signal strength. These are, for each channel, a) the average intensity per pixel across the whole image, b) a signal to noise ratio estimated using the Otsu threshold, and c) a signal to noise ratio estimated as the mean divided by the standard deviation of pixel intensity.

Separately, we developed a set of image quality metrics that require background segmentation. The background areas are initially segmented by mean thresholding on combined nuclear, cytoplasmic, and cell membrane channels, followed by iterative morphological closing to bridge the small and large gaps within the tissue, and then by geodesic active contouring to correct the boundaries (1). The fraction of pixels in the image background is reported, under the assumption that good images should be mostly covered by tissue. We also calculate features measuring background consistency under the assumption that the pixels in the background should be uniform within a channel and conserved across channels. These metrics are the reciprocal of one plus the average coefficient of variation of pixels in image background across all channels, and the fraction of variance accounted for by the first principal component of pixels in the background across all channels as metrics.

Finally, we developed a set of image quality metrics that require cell segmentation. These are calculated for each channel separately, and consist of the average per cell ratios of the cell total intensity divided by the background intensity, and the average nuclear intensity divided by the average cell intensity.

```
{
    "Image Information": {
        "Number of Channels": 59
    },
    "Image Quality Metrics not requiring image segmentation": {
        "Signal To Noise Otsu": {
            "BCL2": 58.071456540532736,
                                        ...
            "aSMA": 78.97842219310057
        },
        "Signal To Noise Z-Score": {
            "BCL2": 0.1929455808042546,
                                        ...
            "aSMA": 0.3095435031673124
        },
        "Total Intensity": {
            "BCL2": 317424198,
                                        ...
          "aSMA": 115849305
        }
    },
    "Image Quality Metrics requiring background segmentation": {
        "1/AvgCVBackground": 0.43595480965432865,
        "Fraction of Pixels in Image Background": 0.624532242428036,
        "FractionOfFirstPCBackground": 0.12844002683241978
    },
    "Image Quality Metrics that require cell segmentation": {
        "Channel Statistics": {
            "Average per Cell Ratios": {
                "BCL2": {
                    "Cell / Background": 12055.829481928306,
                    "Nuclear / Cell": 0.5628348752487062
                },
                                                ...
                "aSMA": {
                    "Cell / Background": 1973.151343042165,
                    "Nuclear / Cell": 0.24329631845879737
                }
            }
        },
        "Fraction of Image Occupied by Cells": 0.1768165219813255,
        "Number of Cells": 22932,
        "Silhouette Scores From Clustering": {
            "Cluster with Max Score": 2,
            "Max Silhouette Score": 0.5353002691007882,
            "Mean-All": {
                "2": 0.5353002691007882,
                                                ...
                "9": 0.4226864392829791
            }
        }
    },
    "Segmentation Evaluation Metrics": {
        "Cell Not Including Nucleus (cell membrane plus cytoplasm)": {
            "1/(AvgOfWeightedAvgCVMeanCellIntensitiesOver1~10NumberOfClusters+1)": 0.29725267801545874,
            "AvgOfWeightedAvgFractionOfFirstPCMeanCellIntensitiesOver1~10NumberOfClusters": 0.2406344017535415,
            "AvgSilhouetteOver2~10NumberOfClusters": 0.3106214242931873
        },
        "Matched Cell": {
            "1-FractionOfBackgroundOccupiedByCells": 0.953088759209949,
            "1/(AvgCVForegroundOutsideCells+1)": 0.13319560976697725,
            "1/(ln(StandardDeviationOfCellSize)+1)": 0.143430457995647,
            "FractionOfCellMaskInForeground": 0.8343051764767412,
            "FractionOfFirstPCForegroundOutsideCells": 0.24637825061816795,
            "FractionOfForegroundOccupiedByCells": 0.3928937614499672,
            "FractionOfMatchedCellsAndNuclei": 1.0,
            "NumberOfCellsPer100SquareMicrons": 0.16909543741343305
        },
        "Nucleus (including nuclear membrane)": {
            "1/(AvgOfWeightedAvgCVMeanCellIntensitiesOver1~10NumberOfClusters+1)": 0.278243529857736,
            "AvgOfWeightedAvgFractionOfFirstPCMeanCellIntensitiesOver1~10NumberOfClusters": 0.21002950986855257,
            "AvgSilhouetteOver2~10NumberOfClusters": 0.2812478574817201
        },
        "QualityScore": -0.08779849034090073
    }
}
```


Supplementary Figure 2. Image Quality Metrics produced by SPRM.

Derived Multichannel Summary Images

SPRM produces three derived images from the multichannel image that do not rely on the cell segmentation. The first is created by finding the principal components of the individual pixel intensities in all channels and assigning the top three to red, green and blue channels in a new image that provides a summary of the ways in which the channel intensities change across the image (an example is shown in Supplementary Figure 3). The second is generated similarly but using non-negative matrix factorization. The third is generated by clustering the pixels into a specified number of clusters (default 3) using all channel intensities and the k-Means algorithm. An indexed image is then created in which the index for each pixel contains its cluster number.

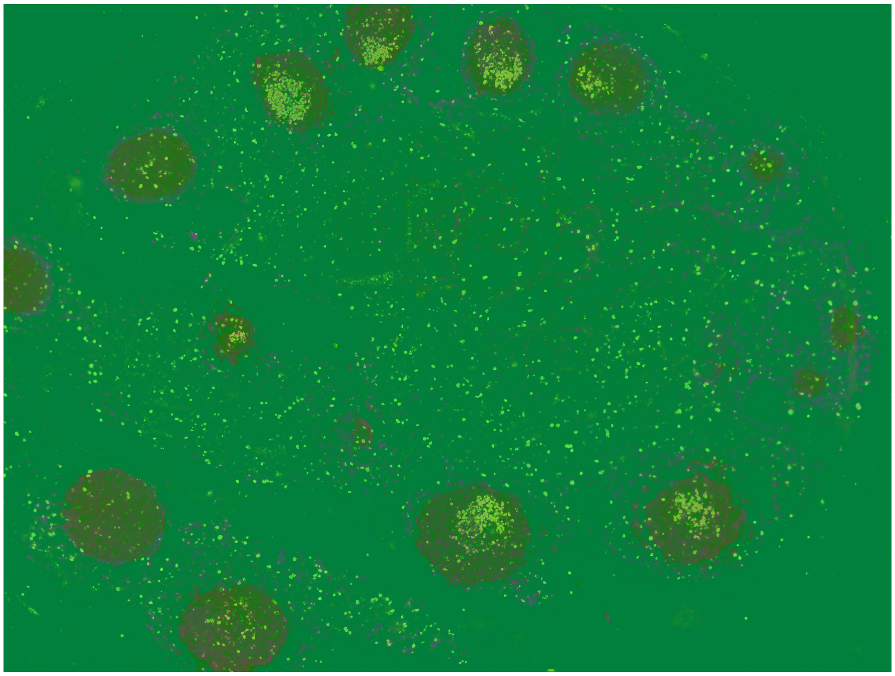

Supplementary Figure 3. Channel Principal Component Analysis on the matrix of pixel intensities for each channel. The first three components are assigned to red, green and blue. (From HuBMAP image doi:10.35079/HBM279.TQRS.775)

Cell Adjacency Matrix

Using just the mask image, SPRM produces a 2D cell adjacency matrix given a user-specification of how far apart cell boundaries may be for them to still be considered neighbors. This is constructed by dilating cell boundaries for a given number of iterations. A sparse square 2D matrix is used to store the distance relationship between all pairs of cells (with zeroes for non-neighboring cells). An example adjacency graph (for a fairly sparse tissue) is show in Supplementary Figure 4 (further adjacency analysis is described below).

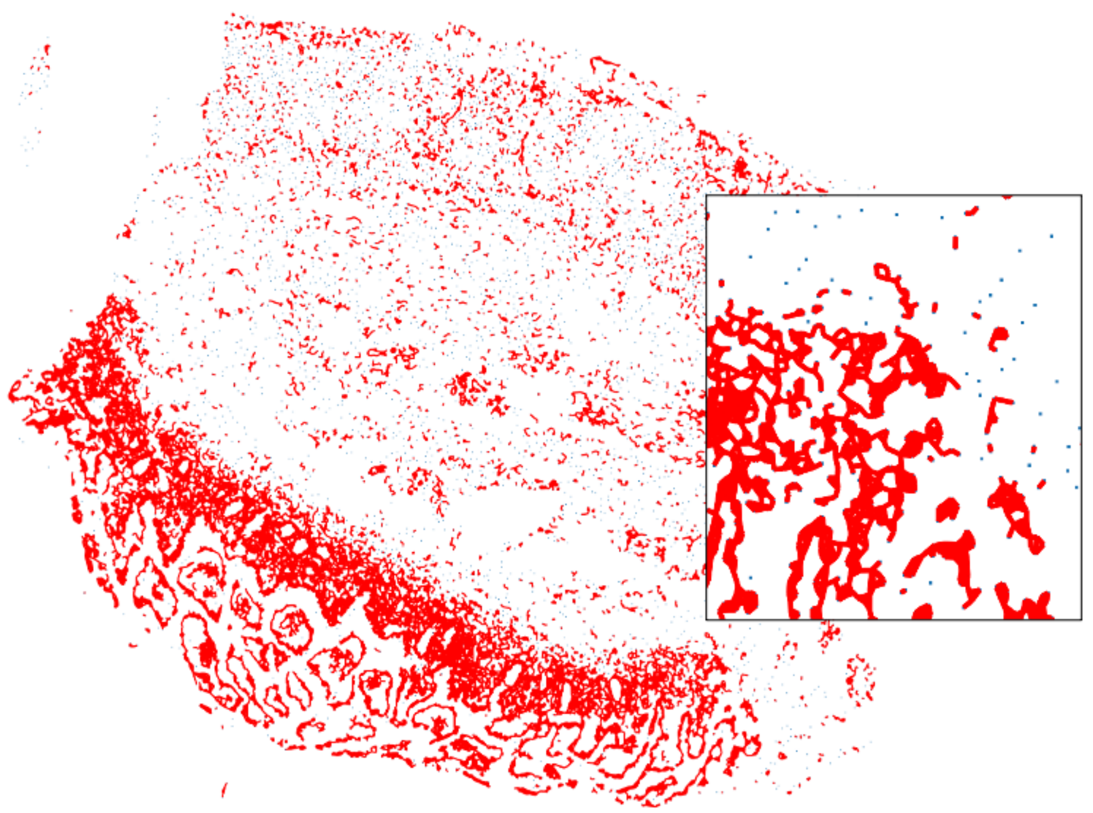

Supplementary Figure 4. Example cell adjacency graph. Cells are represented by points and cells for which any boundary pixels are within a specified distance (in this example, five pixels) of each other are connected by a line. (From HuBMAP image doi:10.35079/HBM899.KTQM.246)

Cell Features

We developed SPRM with a primary goal of producing quantitative descriptions of cell properties in tissue images, and to provide a basis for visualizing, comparing and further analyzing tissue images. We have implemented a large set of features that capture diverse properties of cells in tissues, including content of specific markers (e.g., proteins), spatial distribution of those markers within cells, and cell shape.

SPRM begins by removing edge cells and converting channel intensities into signal-to-noise ratios. Since signal intensities in many imaging modalities are in arbitrary units (and are rarely calibrated to the number of target molecules), this provides a common frame of reference for comparing intensities across different acquisition runs or modalities.

Channel intensities: A core functionality of SPRM is to produce features for each “cell component”, that is, whole cell, whole nucleus, cell boundary only and nuclear boundary only. For each component, the total intensity and mean intensity of each channel is calculated.

Channel textures: Texture features to describe the subcellular patterns are (optionally) calculated using the greycomatrix and greycoprop functions from scipy. This provides 6 properties: contrast, dissimilarity, homogeneity, angular second moment, energy and correlation, for each channel for each cell.

Channel covariances: For each cell, and for each cell component within that cell, the covariance matrix of all channels over all pixels in that component is calculated. This provides an efficient way of comparing the spatial relationships between cells.

Cell shape: Cell shape descriptors are derived from the parametric outlines for each cell (2). Each cell is first rotated to vertically align the major axis of the cell by finding the largest eigenvector from the covariance matrix of horizontal and vertical pixel coordinates. Additionally, the skewness of the horizontal pixel coordinates of the rotated cell is determined to “flip” the cell so that its ‘heavy end’ is on the right side. Afterwards, interpolation along the outline of each cell is done to produce a user-specified number of points (default 100) that trace the shape of each cell. Lastly, PCA is used on the parametric outlines with the number of principal components being the same as the number of points. The principal component coefficients are used as the cell shape descriptors for each cell.

Cell Clustering to estimate cell types

SPRM performs k-means clustering using various subsets and combinations of the above features. Clustering is done separately for each type of cell component (cell, nucleus, cell boundary, nuclear boundary) using total intensity alone, mean intensity alone, and covariance matrices alone. Clustering is also done (separately) using the concatenated mean intensities for all cell components, the concatenated texture features for all channels, and the cell shape features. The number of clusters can be either a user-specified number, or determined automatically using the maximum silhouette score from a wide range of different numbers of clusters. For each clustering, an indexed image is created with the pixels for each cell assigned that cell’s cluster number.

A summary file is created containing a matrix of cluster numbers with columns for each feature set/clustering and rows for each cell. In addition, the cluster centers for each clustering are used to find a set of markers, which are the major independent contributors or features to the clusters. These markers are essentially a ‘legend’ to accompany the clustering results by showing which specific features contribute the most to the clustering.

The most important feature category is those involving channel/marker intensities, and an important issue when calculating cell intensities for 2D tissue images is whether to report total or mean intensity per cell. This issue arises because only a slice of each cell is present in the image and the volume present may vary significantly from cell to cell. Supplementary Figures 5A and 5B show an example image in which either total or mean intensities of all channels for each cell were used as input to k-means clustering (with number of clusters chosen to maximize silhouette score) and cells are colored by their cluster. The clusters presumably correspond (at least approximately) to different cell types. Similarity scores (Supplementary Table 2) indicate that the two clusterings are modestly different. The results suggest that mean intensity is preferable since the resulting clusters are more spatially cohesive (e.g., cells surrounding vessels and tubules are more consistently labeled).

Subcellular distributions of a given protein often vary from cell to cell and cell type to cell type. We next asked whether gross characterization of subcellular distributions by separately quantifying intensities in major cell components (cell, nucleus, nuclear membrane, cell membrane) provides further resolution in cell clustering (Supplementary Figure 5C). However, the clusters incorporating subcellular distribution are quite similar to those for mean cell intensity (similarity score = 96.15) indicating that, at least for this tissue and markers, the gross characterization does not provide much additional information. However, a number of features have been described for sensitive characterization of subcellular distributions (4). Texture features are of particular value for this purpose, and these can be calculated by SPRM for each cell and each channel. However, these are most useful for images with pixel sizes in the 0.1 micron range. Since CODEX images have larger pixel sizes/lower resolution (0.377 microns), we explored the use of covariance matrices as an alternative metric for subcellular differences in multichannel images. That is, we asked whether measuring the pairwise pixel-to-pixel variation in channel intensities for individual cells would reveal differences between marker spatial arrangements (e.g., CD4 being uniformly colocalized with cadherin in some cells but in separate patches in other cells). For each cell, we calculated the pixel-wise covariance matrix of all channels; comparison of the covariance matrices reveals whether colocalization relationships are conserved across cells. Supplementary Figure 5D shows clustering results that are similar to

Supplementary Figures 5A-C, indicating that for this specific dataset the colocalization relationships do not reveal major differences among or within the cell types.

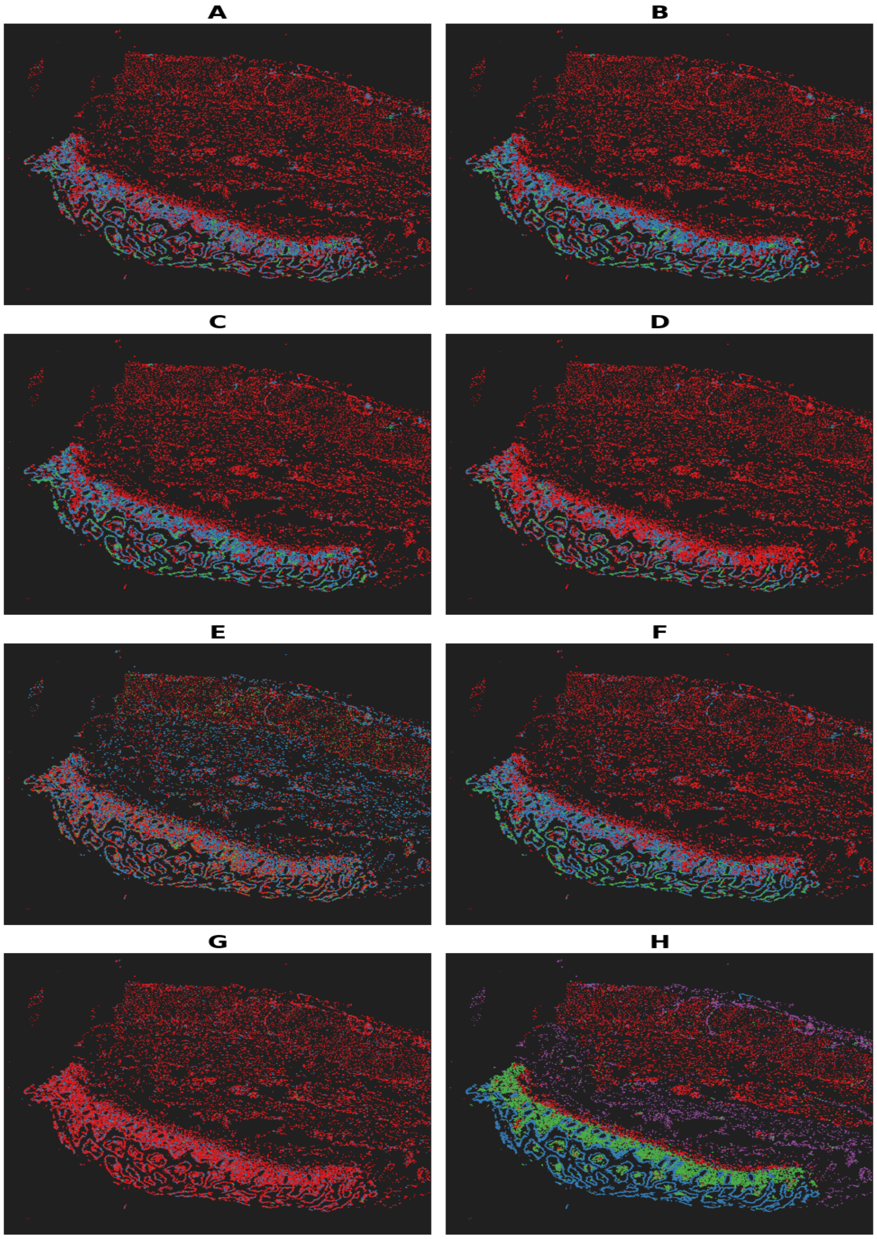

Supplementary Figure 5. Comparisons of cell clustering using different features. A small intestine image from HuBMAP (doi:10.35079/HBM899.KTQM.246) is shown in which each cell is colored by its cluster as defined by k-means clustering using a particular feature set: (A) total intensity per cell, (B) mean intensity per cell, (C) mean channel intensities across four cell components (cytoplasm, nucleus, cell boundary, nuclear boundary), (D) channel covariance, (E) cell shape (unnormalized), (F) normalized cell shape, (G) t-SNE embedding of all features, and (H) UMAP embedding of all features. The number of clusters was chosen in each case to maximize the silhouette score.

Supplementary Table 2. Similarity scores between pairs of cell cluster assignments using different feature sets. The letters in parentheses refer to panels in Supplementary Figure 5.

Comparison	Similarity Score
Total Cell Intensity (A) vs. Mean Cell intensity (B)	77.56
Mean Cell (B) vs. Mean All Compartments (C)	96.15
Mean Cell (B) vs. Channel Covariance (D)	78.44
Mean Cell (B) vs. Cell Shape and Size (E)	43.21
Mean Cell (B) vs. Cell Shape (F)	64.32
Mean Cell (B) vs. t-SNE embedding (G)	75.12
Mean Cell (B) vs. UMAP embedding (H)	47.16
Cell Shape and Size (E) vs. Cell Shape (F)	52.82
t-SNE embedding (G) vs. UMAP embedding (H)	47.22
Channel covariance (D) vs. UMAP embedding (H)	42.10

In addition to differences in marker content, cells may differ in shape and size and CODEX images have sufficient pixel resolution to allow analysis of the shapes of individual cells. SPRM therefore provides shape vectors that capture cell shape in a manner allowing comparison between cells (as discussed in the Methods, this is done by aligning the major and minor axes of all cells and representing shape by evenly-spaced polygonal points along the cell boundary). The vectors capture both cell size and shape, but can also be normalized to consider shape alone (in part this may compensate for variation in apparent size due to differences in the vertical (z axis) positioning of each cell within the 2D tissue slice). Considering both cell size and shape (Supplementary Figure 5E), a quite different clustering occurs (the similarity to B is only 0.43) with a band of cells appearing near the top of the image. This is due to cell size differences, since it disappears considering only cell shape (Supplementary Figure 5F).

When all of the features are considered together, even further splitting of the three main clusters is observed. The clustering by t-SNE (Supplementary Figure 5G) is roughly

similar to clustering by mean intensity (score 0.75) but clustering by UMAP (Supplementary Figure 5H) reveals an additional cell layer similar to, but wider than, that seen in Supplementary Figure 5E. The significant difference between the UMAP clustering and the mean intensity clustering (similarity score 0.47) illustrates the potential value of including diverse cell features for characterizing tissue structure.

Cell Subtype Assignment

A number of approaches have recently been described for assigning cell types to individual cells in multiplexed proteomics images (5-10). These typically involve comparison of feature vectors with expert-provided cell type labels. Since SPRM calculates many distinct categories of individual cell features, it potentially provides useful information for cell type assignment. To explore this, SPRM can accept an externally-supplied set of cell type assignments (e.g., created by expert labeling or a trained cell type predictor) and compare them to the results of clustering using various SPRM feature combinations. This is useful in two ways. In the context of projects such as HuBMAP, it can be used for determining how well a trained machine learner applies to a particular image or set of images (i.e., how well it generalizes across diverse image collections that may be different from the ones it was trained on). It also provides potentially higher resolution definition of cell types (e.g., novel subtypes of a known cell type).

To illustrate this capability, Supplementary Figure 6 shows cell subtype assignments produced by comparing provided cell types with clusters produced from various SPRM feature sets. Supplementary Figure 6A shows an image (a slightly modified version of the image described by Hasanaj et al [12]) in which cells are colored by their cell type provided externally by Cellar. (Note that the unsupervised pixel coloring in Supplementary Figure 3 roughly captures the B and T cell patterns.) To analyze the relationships between these annotated cells and the features generated by SPRM, the SPRM feature sets were concatenated, UMAP-transformed, and then clustered into various numbers of clusters (similar to the analysis in Supplementary Figure 5 except that the number of clusters was allowed to vary; see Methods). For each number of clusters, each cluster was assigned to the cell type with which it had the highest fraction match, and the number of clusters that gave the closest agreement with the provided cell types was chosen. The sum of the matches provides an agreement score between the cell labels and the clustering results.

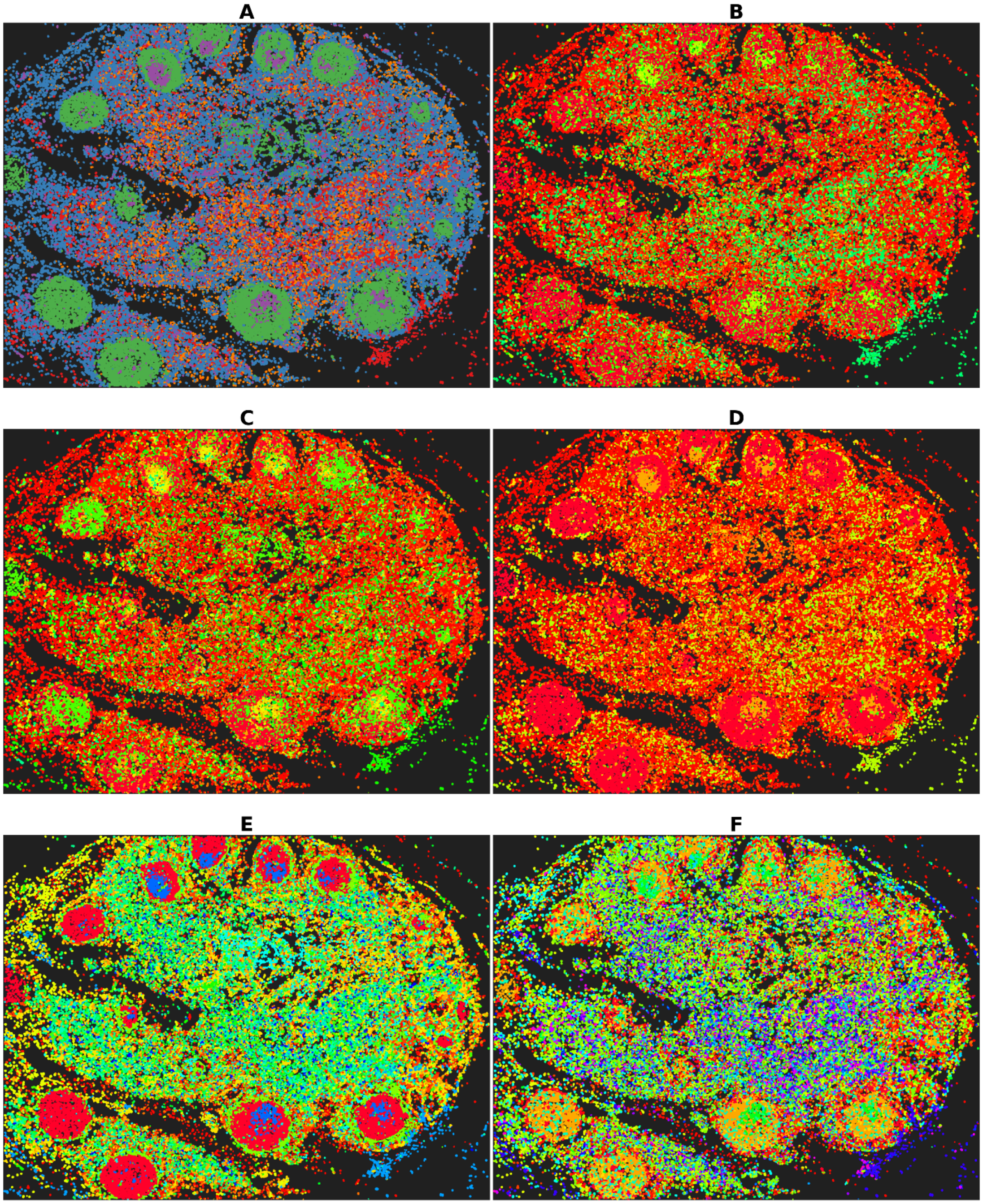

Supplementary Figure 6. Comparison of cell subtyping using different feature sets. A lymph node image from HuBMAP (doi:10.35079/HBM279.TQRS.775) is shown colored by provided cell types and clustering using various combinations of SPRM features. (A) Cell types assigned using Cellar labeling (cytotoxic T cells (red), proliferating T cells (purple), CD4-positive T cell (orange), B cells (green), other cells (blue)). Cells were also clustered using (B) normalized shape features (similarity to A: 38.4%), (C) marker covariance (similarity to A: 53.0%), (D) marker mean cell intensity (similarity to A: 63.2%), (E) UMAP embedding of all features (similarity to A: 66.9%), and (F) t-SNE embedding of all features (similarity to A: 41.6%).

Cells colored by their cluster number are shown for different feature combinations in Supplementary Figures 6B-F. Only the clustering using UMAP embedding (Supplementary Figure 6E) shows reasonable agreement with the provided cell types.

Using this optimal clustering, a unique label (a cell subtype) was generated for each cell by combining the original cell type label and its cluster number (combinations with a small number of cells were merged into an “other” category). Supplementary Figure 7 shows the resulting subtypes for each of the cell types. These often differ in their spatial arrangement. For example, the main subtype of B cells (red in Supplementary Figure 7B) that surrounds the tight clusters of cytotoxic T cells (green in Supplementary Figure 7D) is distinct from the B cells (blue in Supplementary Figure 7B) peripheral to those clusters and also distinct from the minor B cell subtype that is more diffusely distributed (green in Supplementary Figure 7B). Similarly, the subtype of tightly clustered cytotoxic T cells (green in Supplementary Figure 7D) is distinct from the more diffuse subtype (red in Supplementary Figure 7D). Peripheral proliferating T cells (Blue in Supplementary Figure 7C) are distinct from the diffuse central proliferating T cells (red in Supplementary Figure 7C). This type of result can provide potential new directions for exploring tissue composition.

Inferring multimodal intensity allocations

In some experiments, images are taken in two modalities that differ in their spatial resolution. In that case, the larger pixels of the lower resolution modality may cross cell boundaries and complicate allocating the channel intensity of each pixel among cells. During acquisition of the lower resolution image, a pixel that covers two cells, one of which is positive for a marker and another that is negative, would acquire an area-weighted average of the positive and negative intensities. Under the assumption that markers are roughly evenly distributed across each cell, we can estimate how much of teach of the low resolution pixel intensities should be allocated to each of the cells it overlaps. If a second modality image is provided, SPRM will perform this estimation and per-cell features will then be calculated for the second modality.

Interface to HuBMAP Portal

When used in HuBMAP pipelines, the files produced by SPRM are linked to the HuBMAP Portal so that they can be viewed or downloaded. Extensive visualization options are provided through Vitessce (3), including high-resolution interactive viewing of channel intensities and cell cluster assignments using the various approaches described above. Individual results files can be viewed through the portal, and full sets of files downloaded via Globus.

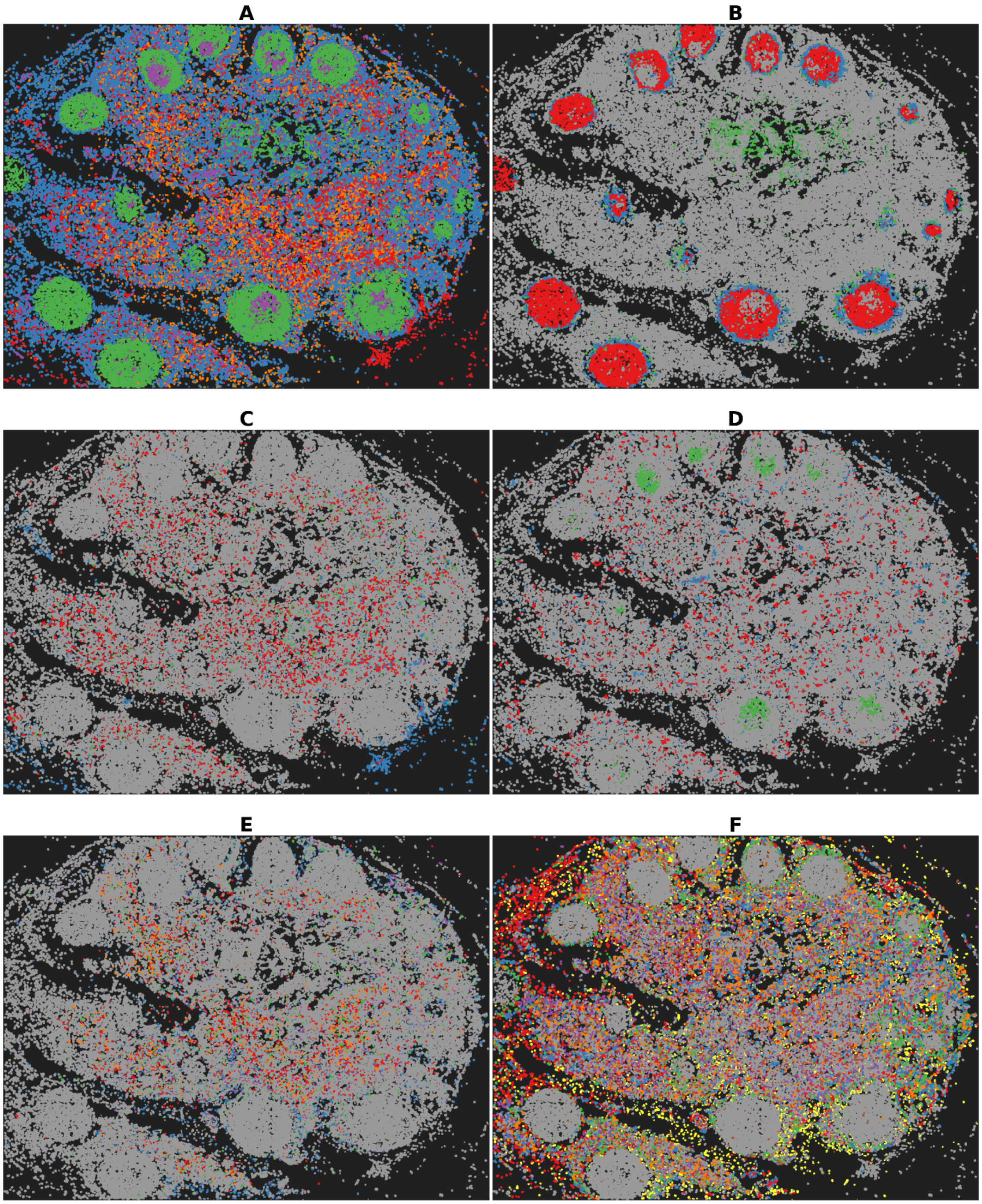

Supplementary Figure 7. Identification of cell subtypes using SPRM features. Results are shown for the same tissue image used in Supplementary Figure 6. Starting cell types (A) were assigned using Cellar labeling (cytotoxic T cells (red), proliferating T cells (purple), CD4-positive T cell (orange), B cells (green), other cells (blue)). Cells were also clustered using UMAP embedding of all SPRM features and then assigned to subtypes of the starting cell types. (B) B cell subtypes, (C) Cytotoxic T cell subtypes, (D) proliferating T cell subtypes, (E) CD-4 positive T cell subtypes, and (F) other cell type subtypes.

SPRM Package

As an alternative to using the full SPRM program, individual analysis described above can be performed using specific modules in the SPRM package (Supplementary Table 3). After installing the “sprm” package, this approach begins by calling the preprocessing module to read the input files and create an object containing the image and various associated data. Any of the further analysis modules listed can then be called (note that the Clustering module requires calling the Cell Features module first).

Supplementary Table 3. sprm Package Modules

1. **Preprocessing** (Required) - Load images, extract ROIs, do quality control
2. **Segmentation Evaluation** (Optional) - Assess segmentation quality
3. **Shape Analysis** (Optional) - Extract cell shape features
4. **Spatial Graphs (Cell Neighborhood Graphs)** (Optional) - Compute spatial relationships
5. **Image Analysis (Pixel Level Analysis)** (Optional) - NMF, superpixels, channel PCA
6. **Cell Features** (Required for clustering) - Intensity & texture features
7. **Clustering** (Analysis) - Multiple clustering methods

Example analyses using SPRM for multiple datasets

Multiple-dataset analysis

SPRM produces separate results files for each provided dataset. To examine aspects of the variation from dataset to dataset and tissue to tissue, we performed analyses using the SPRM outputs for HuBMAP datasets from five tissues. These were spleen, lymph nodes and thymus, collected by the University of Florida HuBMAP Tissue Mapping Center, and small and large intestine, collected by the Stanford University Tissue Mapping Center. When making comparisons across tissues, only the five channels in common across all datasets (CD11c, CD21, CD4, CD8, Ki67) were used.

Image and Segmentation Quality Metrics

Supplementary Figure 8 illustrates the variation in segmentation quality score for each image across tissues. Spleen, thymus and lymph nodes all show better scores in

general compared to small and large intestine. However, there is significant variation among both thymus and lymph node samples.

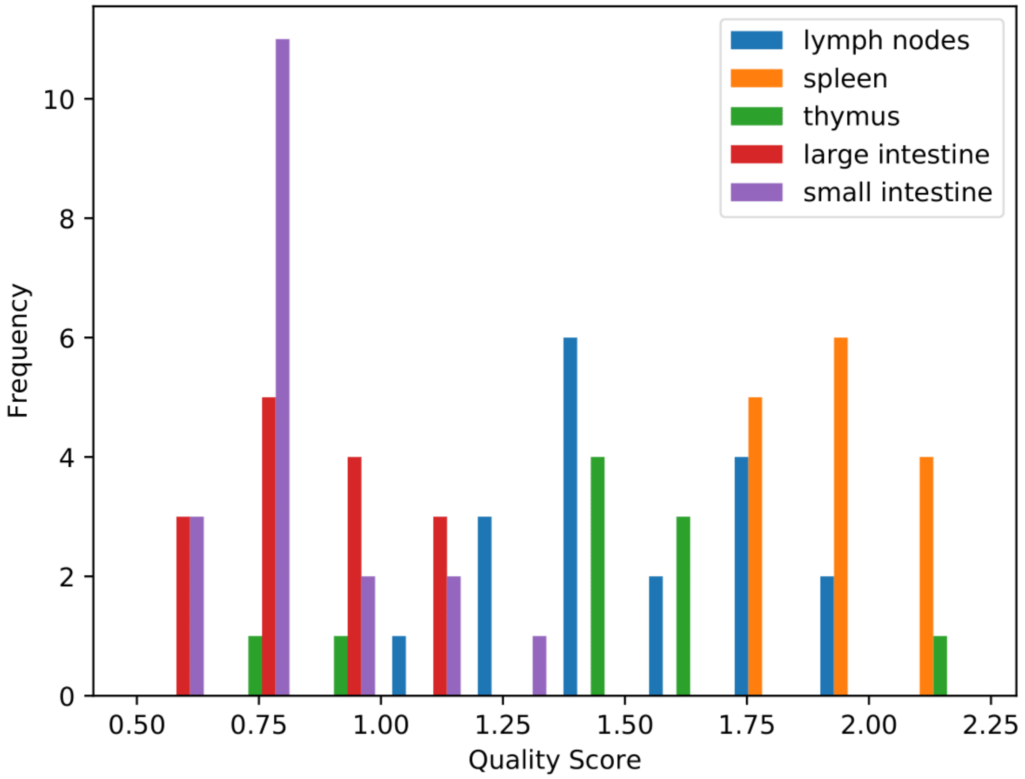

Supplementary Figure 8. Histogram of segmentation quality scores of individual images from different tissues.

We also examined the variation in the image quality metrics between samples. These metrics are mainly designed for comparison from sample to sample of the same tissue, but a subset are useful for comparison between tissues. As an illustration, Supplementary Figure 9 shows the fraction of image covered versus the average signal-to-noise ratio (as measured by mean-to-standard-deviation ratio) across the five common markers. The results show that the thymus images contain both the highest signal-to-noise and the most variation in signal-to-noise (the high variation is similar to that seen for thymus in Supplementary Figure 8).

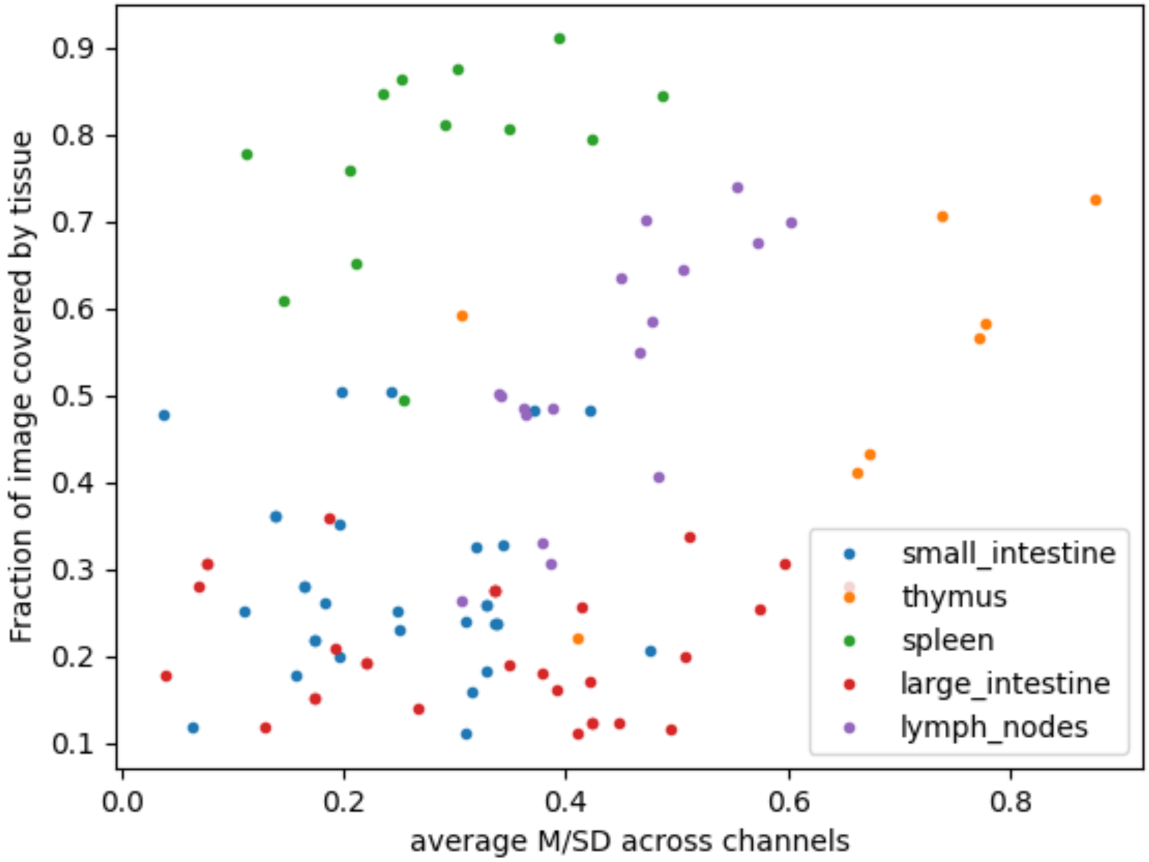

Supplementary Figure 9. Example analysis of variation in image quality metrics across different tissues. This analysis compares two major types of metrics produced by SPRM. Each symbol shows the resulting components for one image.

Cell Shape Analysis

Previous studies have explored the differences in cell shape that either occur naturally or are associated with a specific process (13-15). Most attention has been paid to shape variation in cultured cells, which have fewer constraints on shape than cells in tissue. In order to examine the major modes of cell shape variation within and across datasets and tissues, the standardized cell shape descriptors produced by SPRM were aggregated from 99 CODEX datasets across five different tissues and principal components analysis was performed to get an embedding consisting of just the first two principal components.

Cell shape analysis is complicated by whether to include cell size or to strictly focus on shape (by normalizing shape descriptors to constant size). Supplementary Figures 10A and 10B show results of Kmeans clustering cells both ways. Supplementary Figure

10C shows shapes for example marked cells at various positions in Supplementary Figures 10A and 10B). As might be expected given the constraints imposed by tissue organization, most cells (represented by cells d-f) are roughly symmetric and square or hexagonally shaped.

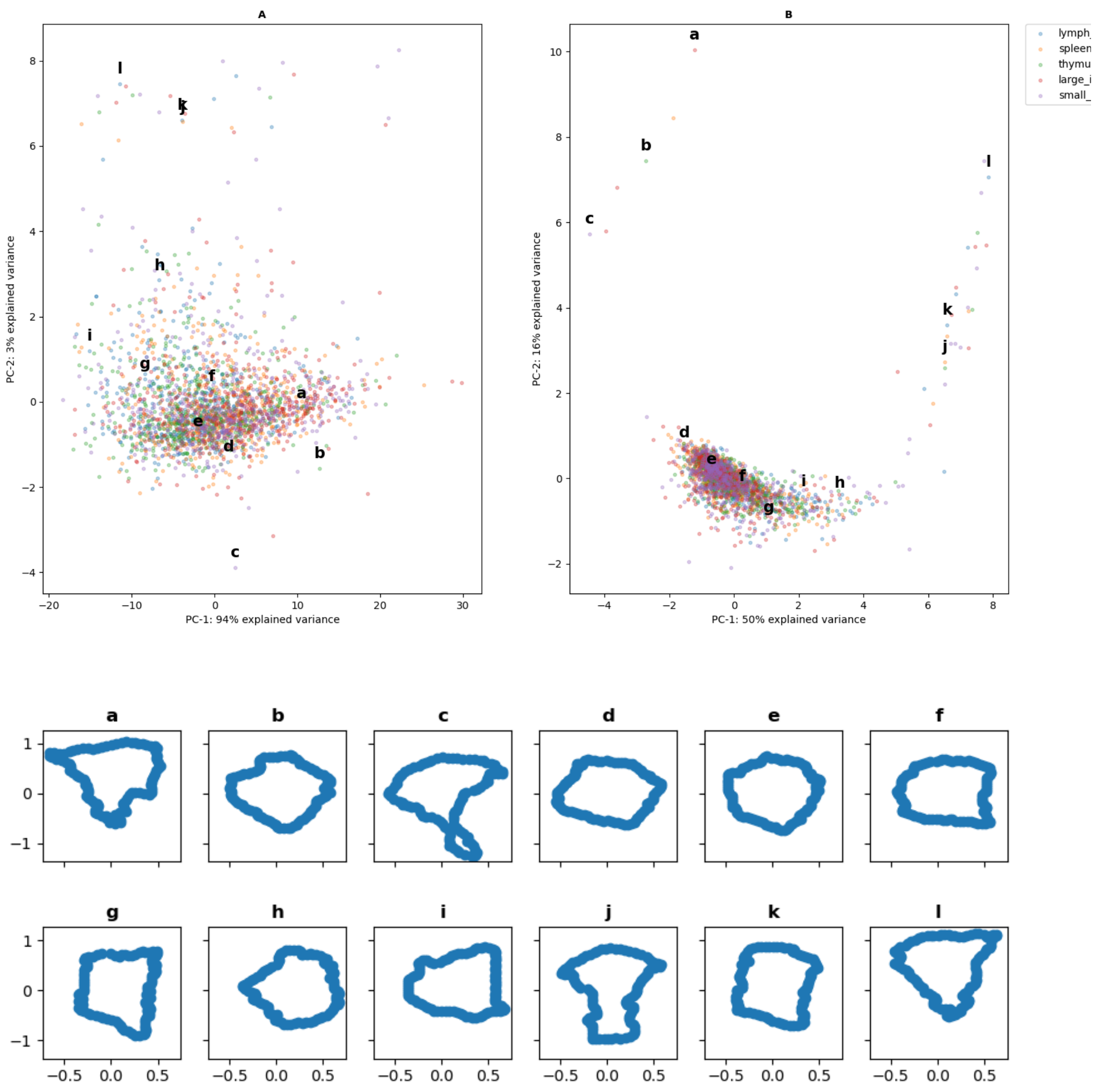

Supplementary Figure 10. Principal component analysis of cell shape. Points indicate the first two principal components of individual cell shape either normalized to the same size (A) or unnormalized (B); color indicates the tissue of origin. Panels a-l show the normalized cell shapes corresponding to labeled cells in panels A and B.

Cell Adjacency Analysis

An additional property of tissue images is the proximity of cells to each other. This property can be described by a matrix showing the distance of all cells to each other. However, the size of such a full matrix would be the square of the number of cells, and distances between pairs of cells that are far apart are not particularly useful. SPRM therefore generates a sparse matrix containing entries only for pairs of cells that are within a specified distance of each other. This matrix has a corresponding cell adjacency graph consisting of a node for each cell and an edge between pairs of nodes with entries in the matrix. Note that an edge does not imply that the two cells are touching, only that they are close to each other (within the threshold distance).

As an illustration, we calculated a node degree histogram (frequently referred to as a node degree spectrum) from the cell adjacency matrix/graph of each of our images. This spectrum is generated by counting the number of cells that have a particular number of cells connected to them. Principal components analysis was then done on the spectra from all images to characterize the variation in cell adjacency among the images

As shown in Supplementary Figure 11A, images separate into four clusters, whose typical spectra are shown in Supplementary Figure 11B. Small and large intestine images are primarily found in the first two clusters, with cluster 1 images having many more cells with 4 or more neighbors. This is illustrated in Supplementary Figures 11C and D, which shows the small intestine images closest to the centroids of clusters 1 and 2.

Discussion

Recent growth in various technologies for multiplexed cell and tissue imaging provide an opportunity for detailed analysis of the spatial relationships within cells and between cell types within tissues. A number of projects, such as HuBMAP, are using these technologies to construct large collections of images for different tissues using different technologies. This provides a significant opportunity for detailed analysis of the spatial

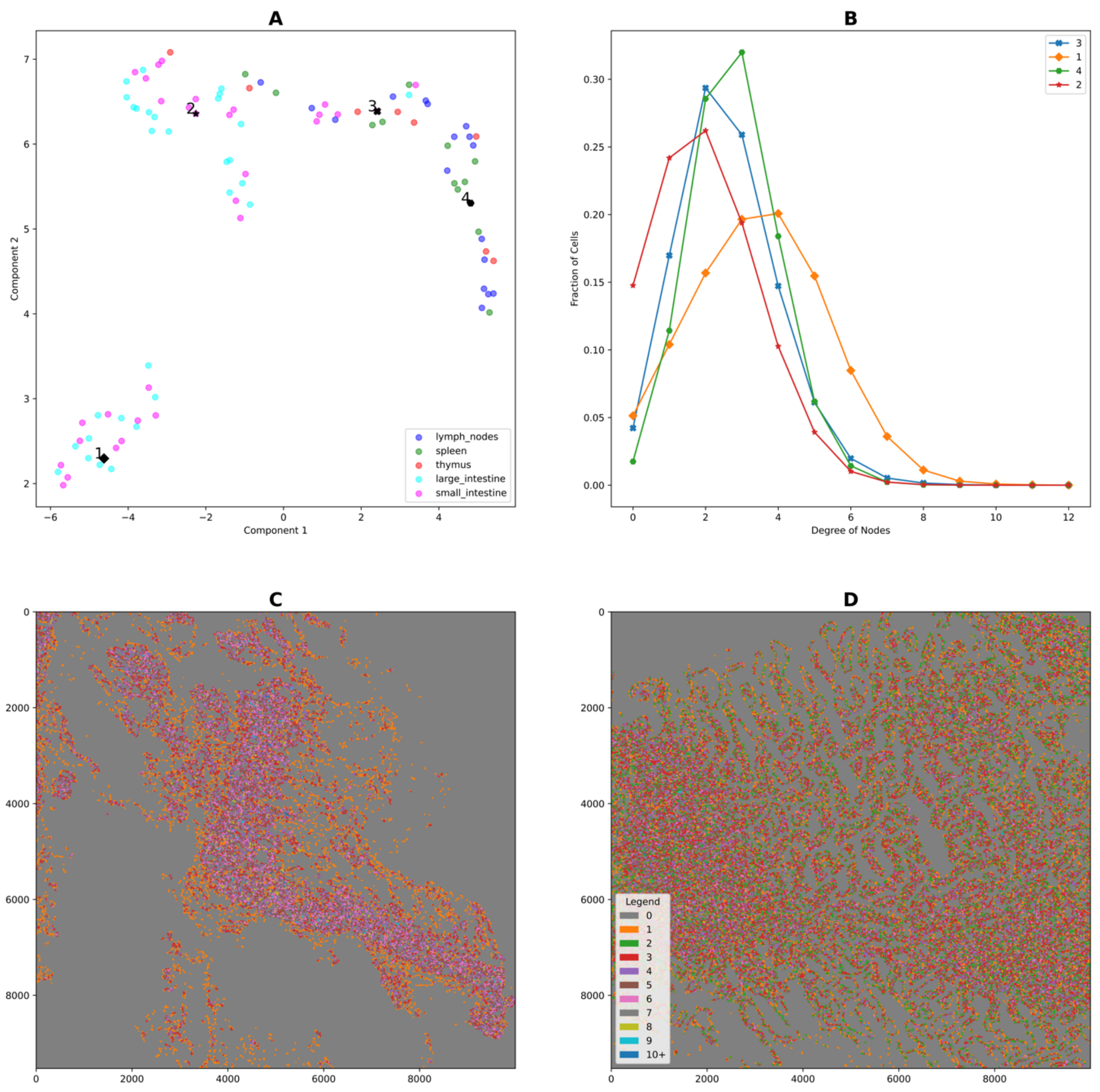

Supplementary Figure 11. Comparison of cell adjacencies. A) The first two principal components of the node degree vectors for all images are shown, colored by tissue (each symbol is one image). The images were clustered into four numbered clusters. B) The node degree vector for the image closest to each cluster centroid is shown for each tissue. C) Image closest to the centroid of cluster 1, with each cell colored by its degree. D) Image closest to the centroid of cluster 2 (color legend applies to both images).

relationships within cells and between cell types within tissues and a corresponding need for additional analysis software.

We have therefore developed SPRM as part of the HuBMAP image analysis pipelines. The goal is to enable subsequent visualization and comparison of diverse cell properties

within and between imaging modalities and tissues. It can readily be used in studies outside of HuBMAP for any multiplexed images with corresponding segmentations.

Tissue imaging studies typically produce images with much larger numbers of pixels (often created by stitching together smaller, overlapping tiles) than most fluorescence microscopy studies. This creates both opportunities as well as complications for processing. Images of higher resolution generally contain more cells which in turn takes up more memory. SPRM is optimized for both memory and compute time to handle these large images. Generally, most processes are bounded by the number of cells in the segmented mask and therefore linear in time. If available, SPRM can take advantage of multithreading on high performance computing clusters.

Two novel contributions that may be useful for incorporation into other analysis programs are worth noting . The first consists of features for exploring subcellular variation in marker patterns that are suitable for the moderate spatial resolution typical of multiplexed tissue images. This permits potential identification of different cell states or cell subtypes.

The second relates to assessment of image quality. The advent of imaging projects with large numbers of images also creates a need for means to evaluate the quality of those images and the results obtained from them. To this end, we have developed and implemented a number of metrics that can be used to reflect the expected confidence in results for any given image and to filter images to be included in analysis based upon that confidence. We observed significant variation in both segmentation quality score and image quality metrics among the datasets we examined.

Conclusions

The results shown here demonstrate 1) Inclusion of a diverse set of cell features reveals differences in cell clusters/types beyond those revealed by marker intensities alone (Supplementary Figure 5). 2) When provided with externally-generated assignment of cell types, SPRM can both measure the likelihood that the assignment is applicable to that image, and can identify potential cell subtypes that add further refinement to spatial cell maps (Supplementary Figures 6 and 7). 3) Analysis of signal-to-noise ratios, segmentation quality scores and image quality metrics reveal significant variation among different images from small intestine (Supplementary Figures 8 and 9). The significance of these different cell clusterings and their spatial arrangement will require additional investigation. 4) Detailed analysis of cell shape reveals a continuous range of common shapes from ovoid to polygonal, as well as a number of less frequent shapes (Supplementary Figure 10). 5) Analysis of cell adjacency graphs reveals differences in cell arrangements within and between tissues (Supplementary Figure 11).

References

1. Chen H, Murphy RF. Evaluation of cell segmentation methods without reference segmentations. Molecular Biology of the Cell. 2023;34(6):ar50.
2. Pincus Z, Theriot JA. Comparison of quantitative methods for cell-shape analysis. J Microsc. 2007;227(Pt 2):140-56.
3. Keller MS, Gold I, McCallum C, Manz T, Kharchenko PV, Gehlenborg N. Vitessce: a framework for integrative visualization of multi-modal and spatially-resolved single-cell data. OSF Preprints. 2021;10.
4. Newberg J, Hua J, Murphy RF. Location proteomics: systematic determination of protein subcellular location. Systems Biology. 2009:313-32.
5. Brbić M, Cao K, Hickey JW, Tan Y, Snyder MP, Nolan GP, et al. Annotation of spatially resolved single-cell data with STELLAR. Nature Methods. 2022;19(11):1411-8.
6. Geuenich MJ, Hou J, Lee S, Ayub S, Jackson HW, Campbell KR. Automated assignment of cell identity from single-cell multiplexed imaging and proteomic data. Cell Systems. 2021;12(12):1173-86.e5.
7. Hasanaj E, Wang J, Sarathi A, Ding J, Bar-Joseph Z. Interactive single-cell data analysis using Cellar. Nature Communications. 2022;13(1):1998.
8. Lee E, Chern K, Nissen M, Wang X, Consortium I, Huang C, et al. SpatialSort: a Bayesian model for clustering and cell population annotation of spatial proteomics data. Bioinformatics. 2023;39(Supplement_1):i131-i9.
9. Mongia A, Zohora FT, Burget NG, Zhou Y, Saunders DC, Wang YJ, et al. AnnoSpat annotates cell types and quantifies cellular arrangements from spatial proteomics. Nature Communications. 2024;15(1):3744.
10. Shaban M, Bai Y, Qiu H, Mao S, Yeung J, Yeo YY, et al. MAPS: pathologist-level cell type annotation from tissue images through machine learning. Nature Communications. 2024;15(1):28.
11. Van der Maaten L, Hinton G. Visualizing data using t-SNE. Journal of machine learning research. 2008;9(11).
12. McInnes L, Healy J, Melville J. Umap: Uniform manifold approximation and projection for dimension reduction. arXiv preprint arXiv:180203426. 2018.
13. Johnson GR, Buck TE, Sullivan DP, Rohde GK, Murphy RF. Joint Modeling of Cell and Nuclear Shape Variation. Mol Biol Cell. 2015;26(22):4046-56.
14. Ruan X, Murphy RF. Evaluation of methods for generative modeling of cell and nuclear shape. Bioinformatics. 2019;35(14):2475-85.
15. Ruan X, Johnson GR, Bierschenk I, Nitschke R, Boerries M, Busch H, et al. Image-derived models of cell organization changes during differentiation and drug treatments. Mol Biol Cell. 2020;31(7):655-66.
